# The Development of Sarcoidosis in an Ulcerative Colitis Patient Treated with Vedolizumab: A Case Report and Review of the Literature

**DOI:** 10.3390/clinpract16020044

**Published:** 2026-02-23

**Authors:** John K. Triantafillidis, Konstantinos Malgarinos, Loukas Kaklamanis, Emmanouil Kritsotakis, Victoria Polydorou, Konstantinos Pantos, Konstantinos Sfakianoudis, Agni Pantou, Konstantinos Bramis, Manousos M. Konstantoulakis, Apostolos E. Papalois

**Affiliations:** 1 IBD Unit, “Metropolitan General” Hospital, 264 Mesogeion Avenue, 15562 Athens, Greece; jktrian@gmail.com (J.K.T.); gmalgarinos@yahoo.co.uk (K.M.); 2 Hellenic Society of Gastrointestinal Oncology, 354, Iera Odos str., 12461 Athens, Greece; 3 Department of Pathology, Onassis Hospital, 356 Sygrou Avenue, 17674 Athens, Greece; loukasgka@yahoo.gr; 4 2nd Department of Surgery, Aretaieion University Hospital, School of Medicine, National and Kapodistrian University of Athens, 76 Vas. Sophias Av., 11528 Athens, Greece; ekritsotakis2020@gmail.com (E.K.); kbramis@gmail.com (K.B.); mkonstad@med.uoa.gr (M.M.K.); 5 School of Medicine, European University Cyprus, 6 Diogenous St. Egkomi, Nicosia 1516, Cyprus; v.polydorou@euc.ac.cy; 6 Centre for Human Reproduction, Genesis Athens Clinic, 14-16, 15232 Athens, Greece; info@pantos.gr (K.P.); sfakianosc@yahoo.gr (K.S.); agni.pantos@gmail.com (A.P.)

**Keywords:** ulcerative colitis, sarcoidosis, vedolizumab, biologic agents, inflammatory bowel disease

## Abstract

Background: Ulcerative colitis (UC) and sarcoidosis are chronic inflammatory diseases that share immunological pathways but rarely coexist. The increasing use of biologic agents in inflammatory bowel disease (IBD) has raised concerns regarding paradoxical inflammatory manifestations, including sarcoidosis-like reactions. Case presentation: We report the case of a 63-year-old man with long-standing UC treated with vedolizumab who developed systemic sarcoidosis characterized by bilateral hilar lymphadenopathy, mediastinal and abdominal lymph node enlargement, pulmonary involvement, and erythema nodosum. Extensive diagnostic work-up, including imaging and histopathology, confirmed non-necrotizing granulomatous disease consistent with sarcoidosis, while alternative infectious, malignant, and drug-induced causes were excluded. Vedolizumab was temporarily discontinued, leading to UC relapse, and subsequently reintroduced with rapid clinical remission of UC. Discussion: Sarcoidosis remained clinically and radiologically stable despite vedolizumab re-initiation, suggesting a coincidental association rather than a direct causal relationship. This case highlights the diagnostic challenges and therapeutic dilemmas in patients with immune-mediated diseases receiving biologic therapy. Conclusion: The coexistence of UC and sarcoidosis during vedolizumab therapy is rare. Although causality cannot be established, our findings suggest that vedolizumab may be safely continued in selected patients under close multidisciplinary monitoring.

## 1. Introduction

Ulcerative colitis (UC) is a chronic immune-mediated inflammatory bowel disease (IBD) characterized by continuous mucosal inflammation of the colon, leading to a relapsing–remitting clinical course [1,2]. Although UC is primarily considered a disease of the gastrointestinal tract, increasing evidence supports its systemic nature, with extraintestinal manifestations affecting the skin, joints, hepatobiliary system, and, less frequently, the lungs. These manifestations reflect a broader dysregulation of the immune system rather than isolated intestinal pathology.

Sarcoidosis is a multisystem granulomatous disorder of unknown etiology, characterized histologically by non-necrotizing epithelioid granulomas and clinically by heterogeneous organ involvement [3,4]. The lungs and intrathoracic lymph nodes are most commonly affected, although extrapulmonary manifestations involving the skin, eyes, heart, liver, and nervous system are well recognized. The disease course of sarcoidosis ranges from spontaneous resolution to chronic progressive disease, highlighting the complexity of its immunopathogenesis.

The advent of biologic therapies has significantly transformed the management of IBD, including UC. Tumor necrosis factor alpha (TNF-α) inhibitors were among the first biologic agents introduced and have demonstrated efficacy in both UC and refractory sarcoidosis. Paradoxically, however, anti-TNF-α therapy has been increasingly associated with the development of sarcoidosis or sarcoidosis-like granulomatous reactions, a phenomenon that remains incompletely understood. Proposed mechanisms include immune redistribution, cytokine imbalance, and unmasking of latent granulomatous disease.

Vedolizumab (Entyvio^®^) is a humanized monoclonal antibody targeting the α4β7 integrin, thereby preventing the migration of inflammatory lymphocytes into the gastrointestinal mucosa. Unlike anti-TNF-α agents, vedolizumab exerts a gut-selective mechanism of action and is generally regarded as having a favorable systemic safety profile [5]. Large clinical trials and post-marketing surveillance studies have demonstrated its efficacy and tolerability in both UC and Crohn’s disease, with a relatively low incidence of systemic immunological adverse events [6]. Nevertheless, data regarding its potential association with granulomatous diseases, including sarcoidosis, remain scarce.

Given the rarity of sarcoidosis occurring in patients with UC treated with vedolizumab, the limited data regarding causality, and the potential clinical implications for long-term disease monitoring and therapeutic decision-making, we present this case to contribute to the existing literature [7,8,9,10,11,12]. This report aims to highlight the diagnostic process, management considerations, and follow-up of a patient with concomitant UC and sarcoidosis, while emphasizing the need for cautious interpretation of causality and the importance of multidisciplinary care.

## 2. Case Presentation

A 63-year-old woman with a history of UC diagnosed 15 years earlier was referred to our unit for evaluation of persistent cough and systemic lymphadenopathy. The UC had initially been managed with mesalamine and corticosteroids, followed by azathioprine due to steroid dependency. Owing to a loss of response and intolerance to immunomodulatory therapy, vedolizumab was initiated, resulting in sustained clinical and endoscopic remission.

Approximately three years after the initiation of vedolizumab, the patient developed erythema nodosum, followed by a non-productive cough. Her medical history was notable for a prior appendectomy, previous exposure to azathioprine and corticosteroids, and a documented SARS-CoV-2 infection, all of which were considered during the differential diagnostic evaluation due to their potential immunological relevance.

**Figure 1:**
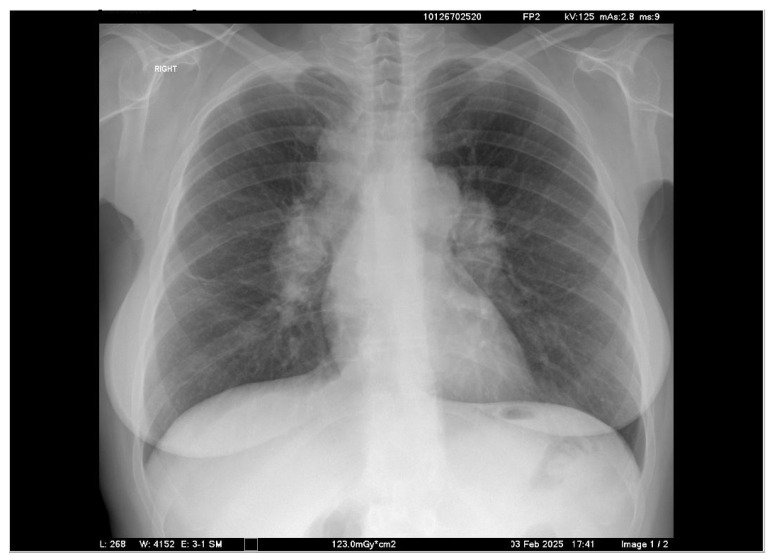
Chest X-ray showing prominent hilar lymphadenopathy.

Chest X-ray showed prominent hilar lymphadenopathy (Figure 1), while chest computed tomography revealed bilateral hilar and mediastinal lymphadenopathy with associated pulmonary infiltrates.

**Figure 2:**
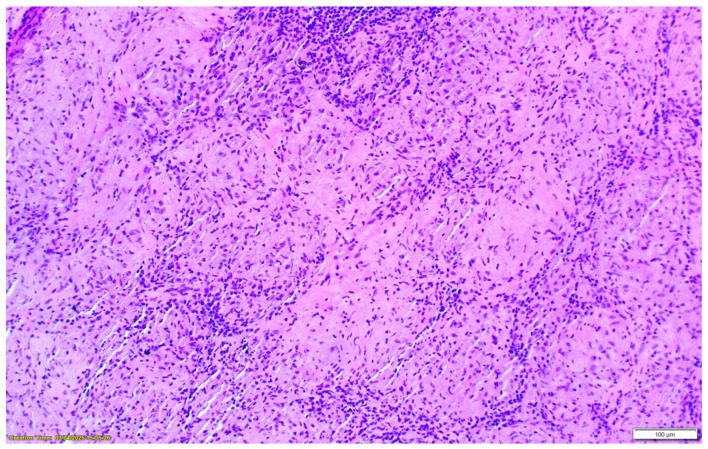
Hematoxylin–eosin staining ×200 magnification of well-formed sarcoid-type granulomas.

**Figure 3:**
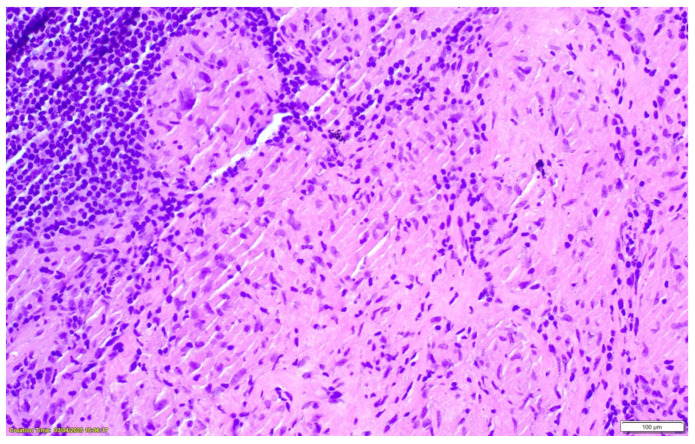
Hematoxylin–eosin staining in ×100 magnification of well-formed sarcoid-type granulomas.

**Figure 4:**
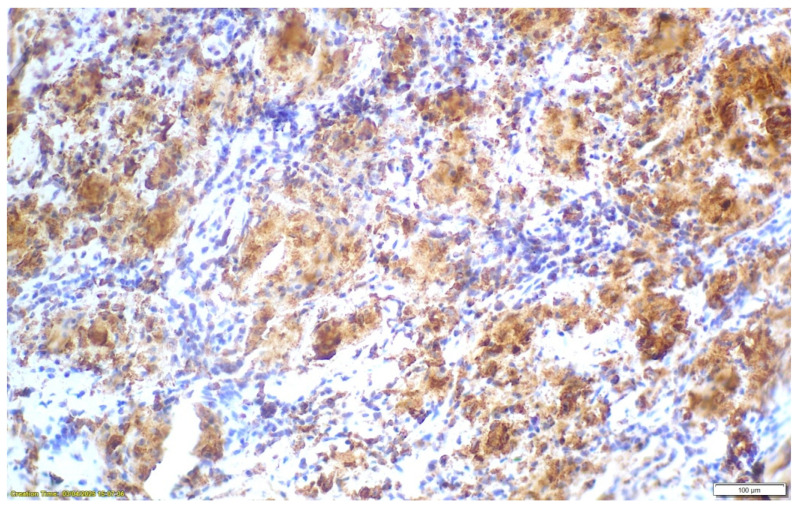
CD68-positive immunostaining reveals the macrophage aggregates forming the sarcoid-type granulomas.

Positron emission tomography–computed tomography (PET-CT) demonstrated metabolically active hilar, mediastinal, and abdominal lymph nodes, raising initial concern for lymphoproliferative disease. Bronchoscopy with transbronchial lymph node biopsy was performed. Histopathological examination revealed well-formed, non-necrotizing epithelioid granulomas with multinucleated giant cells and lymphocytic cuffing. CD68 immunostaining was positive, while special stains for mycobacteria and fungi (including Ziehl–Neelsen, PAS, and Grocott) were negative, and no foreign material was identified. These findings were consistent with sarcoidosis [Figure 2, Figure 3 and Figure 4].

Infectious causes, including tuberculosis and fungal infections, as well as hematological malignancy and drug-induced granulomatosis, were systematically excluded. Pulmonary involvement was classified as radiographic stage I sarcoidosis, limited to lymphadenopathy without significant parenchymal disease. Given the absence of organ-threatening manifestations, preserved pulmonary function, and mild respiratory symptoms, a conservative “watchful waiting” approach was adopted in accordance with current guidelines.

Vedolizumab was temporarily discontinued due to diagnostic uncertainty, resulting in clinical relapse of UC. Following multidisciplinary discussion, vedolizumab was reintroduced, leading to rapid improvement in UC symptoms within two weeks. Importantly, sarcoidosis remained clinically stable, with no worsening of respiratory symptoms or radiological findings during follow-up.

This clinical course—the development of sarcoidosis during vedolizumab therapy, UC relapse after discontinuation, and stable sarcoidosis following re-initiation—suggests a coincidental association rather than a direct causal relationship.

## 3. Discussion

The coexistence of UC and sarcoidosis represents a rare but increasingly recognized clinical scenario, reflecting the complex interplay between systemic immune dysregulation and organ-specific inflammatory disease. In the present case, the diagnostic process was particularly challenging due to the extensive lymphadenopathy and pulmonary findings, which initially raised concern for lymphoproliferative malignancy. This highlights the importance of maintaining a broad differential diagnosis in patients with IBD who develop atypical systemic manifestations.

Sarcoidosis has been reported to occur before, concurrently with, or after the diagnosis of UC, without a consistent temporal relationship with intestinal disease activity [5,6]. In our patient, UC clearly preceded the onset of sarcoidosis, and the latter developed while the intestinal disease was in clinical remission. This temporal dissociation supports the concept that sarcoidosis may arise independently of bowel inflammation and may reflect a shared underlying immune predisposition rather than direct disease extension.

Several mechanisms have been proposed to explain the association between UC and sarcoidosis. Genetic susceptibility appears to play a role, with shared HLA haplotypes reported in patients affected by both conditions [13]. Mendelian randomization studies further support a genetic link between IBD and sarcoidosis, suggesting that immune-mediated pathways common to both diseases may predispose susceptible individuals to their coexistence [14]. Beyond genetics, immune dysregulation represents a central pathogenic feature. Sarcoidosis is characterized by exaggerated Th1-driven granulomatous inflammation, while UC involves complex dysregulation of mucosal immunity. The increased prevalence of immune-mediated inflammatory diseases among patients with IBD reinforces the hypothesis of overlapping immunopathogenic mechanisms rather than a coincidental association [15,16,17].

Regarding the effect of vaccination against COVID-19 infection with mRNA-based COVID-19 vaccination, recent data support that there is no association between vaccination and increased risk of autoimmune diseases, including UC, Crohn’s disease, and sarcoidosis [18]. Concerning the role of exogenous factors, recent data support the existence of an association between glucagon-like peptide-1 receptor agonists and increased risk of sarcoidosis, UC, type 1 diabetes, and systemic lupus erythematosus [19]. A review of 33 studies showed that *Propionibacterium acnes* could be considered a causative factor in sarcoidosis. However, there is a substantial overlap between genetic predisposition and immune dysfunction in the pathogenesis of sarcoidosis, UC, and *Propionibacterium acnes* [20].

The diagnosis of sarcoidosis requires careful exclusion of alternative causes of granulomatous inflammation. In this case, infectious etiologies, including tuberculosis and fungal infections, were systematically excluded through microbiological studies and special histological stains. Hematological malignancy was considered unlikely following histopathological confirmation of non-necrotizing granulomas without evidence of clonal lymphoid proliferation. Drug-induced granulomatosis was also contemplated, given the patient’s exposure to multiple immunomodulatory agents, underscoring the complexity of diagnostic reasoning in such cases.

The role of biologic therapies in the development of sarcoidosis or sarcoidosis-like reactions remains an area of ongoing debate. Anti-TNF-α agents have been most frequently implicated in paradoxical granulomatous reactions, despite their established efficacy in refractory sarcoidosis [21,22,23]. Several mechanisms have been proposed, including altered cytokine balance, incomplete TNF-α blockade, immune redistribution, and unmasking of latent granulomatous disease. These paradoxical effects appear to be more commonly associated with certain agents, such as etanercept, than with monoclonal antibodies.

In contrast, vedolizumab exerts a gut-selective mechanism of action by inhibiting α4β7 integrin-mediated lymphocyte trafficking to the intestinal mucosa. Its limited systemic immunosuppressive effect differentiates it mechanistically from anti-TNF-α agents and provides a theoretical basis for a lower risk of systemic immune-mediated adverse events. Available clinical trial data and real-world studies support the favorable safety profile of vedolizumab, with sarcoidosis not recognized as a typical adverse reaction.

Nevertheless, isolated cases of sarcoidosis occurring in patients treated with vedolizumab have been reported [24,25], although a causal relationship has not been established. In the present case, sarcoidosis developed after prolonged exposure to vedolizumab, raising the question of delayed immune modulation or coincidental disease emergence. Importantly, discontinuation of vedolizumab resulted in relapse of UC, while re-initiation led to rapid clinical improvement without clinical, radiological, or biochemical worsening of sarcoidosis. This clinical course argues against a direct causal association and supports the hypothesis of coincidental coexistence in a patient with underlying immune susceptibility.

The decision not to initiate pharmacological treatment for sarcoidosis was guided by current clinical practice recommendations. The patient exhibited radiographic stage I pulmonary sarcoidosis, limited to lymphadenopathy without significant parenchymal involvement, preserved pulmonary function, and mild respiratory symptoms. In such cases, a watchful waiting approach is considered appropriate, given the potential for spontaneous resolution and the risks associated with systemic corticosteroid therapy. This conservative strategy was further supported by the stability of sarcoidosis during subsequent follow-up.

From a clinical perspective, this case underscores the importance of individualized management and multidisciplinary collaboration. The occurrence of sarcoidosis in patients with IBD receiving biologic therapy should prompt careful evaluation of disease severity, temporal associations, and alternative explanations before attributing causality to a specific agent. When intestinal disease control necessitates continuation or re-initiation of biologic therapy, gut-selective agents such as vedolizumab may represent a reasonable option under close monitoring.

The strengths of this report include the detailed diagnostic work-up, histopathological confirmation of sarcoidosis, and the documented clinical course following re-initiation of vedolizumab. The safe continuation of gut-selective biologic therapy without exacerbation of sarcoidosis provides clinically relevant insight for physicians facing similar therapeutic dilemmas.

However, several limitations must be acknowledged. This is a single case report with limited follow-up, and potential confounding factors, including prior exposure to immunosuppressive therapies, cannot be fully excluded. As such, the findings should be interpreted cautiously and viewed as hypothesis-generating rather than definitive evidence.

Future studies are needed to better elucidate the relationship between biologic therapies and granulomatous diseases, as well as to identify potential biomarkers that may predict susceptibility to paradoxical immune reactions. Until more robust data become available, clinicians should remain vigilant for systemic inflammatory manifestations in patients with IBD and adopt a personalized, multidisciplinary approach to management.

## 4. Conclusions

The present case highlights a rare clinical association between UC and sarcoidosis in a patient treated with vedolizumab and underscores the complexity of immune-mediated diseases in the era of biologic therapy. Although sarcoidosis developed during ongoing vedolizumab treatment, the overall clinical course does not support a clear causal relationship between the drug and the onset of granulomatous disease.

Importantly, the relapse of UC following vedolizumab discontinuation and the subsequent rapid clinical improvement after re-initiation, without evidence of sarcoidosis progression, suggests that the coexistence of these two conditions may reflect an underlying immune predisposition rather than a drug-induced adverse effect. This observation is of particular clinical relevance, as it supports the possibility of continuing gut-selective biologic therapy in selected patients when disease control necessitates ongoing treatment.

From a broader perspective, this case emphasizes the need for careful diagnostic evaluation and cautious interpretation of temporal associations between biologic therapies and systemic inflammatory manifestations. Clinicians should remain aware that sarcoidosis may occur independently of IBD activity and may emerge coincidentally during biologic treatment. Thorough exclusion of alternative etiologies and close multidisciplinary collaboration are essential to guide appropriate management decisions.

Given the limited number of reported cases and the inherent constraints of single-case observations, definitive conclusions regarding causality cannot be drawn. Nevertheless, this report contributes valuable clinical insight by documenting the stable course of sarcoidosis following re-initiation of vedolizumab and highlights the importance of individualized risk–benefit assessment.

Further studies and long-term observational data are needed to better define the relationship between gut-selective biologic agents and granulomatous diseases and to inform evidence-based monitoring strategies in patients with IBD.

## Data Availability

The data presented in this study are available on request from the corresponding author due to privacy restrictions.

## Abbreviations

ACE	Angiotensin-converting enzyme
AZA	Azathioprine
CS	Corticosteroids
CT	Computed tomography
FDG	Fluorodeoxyglucose
IBD	Inflammatory bowel disease
IL	Interleukin
JAK	Janus kinase
PET-CT	Positron emission tomography–computed tomography
TNF-α	Tumor necrosis factor alpha
UC	Ulcerative colitis
VDZ	Vedolizumab
